# Mechanical and physical properties of polyethylene/sour cherry shell powder bio‐composite as potential food packaging

**DOI:** 10.1002/fsn3.2264

**Published:** 2021-03-29

**Authors:** Sahra Farhadi, Majid Javanmard

**Affiliations:** ^1^ Food Technologies Group Department of Chemical Engineering Iranian Research Organization for Science and Technology (IROST) Tehran Iran

**Keywords:** bio‐composite, polyethylene, sour cherry shell, waste valorization

## Abstract

Eco‐friendly composite materials have received more attention in recent years, and research has shown that biostructures have great potential as a solution to meet the needs of sustainability in product design and potential food packaging. In this study, the mechanical, thermal, morphological, water absorption properties and water vapor permeability of bio‐composites based on polyethylene and sour cherry shell powder (SCS) (0% –7.5%) have been investigated. It was observed that 2.5% of sour cherry shell increased elastic modulus and tensile strength and improved mechanical properties. Composites without adding sour cherry shell show 1.28% water absorption. Decreased water absorption was observed for treated composites containing 2.5% of sour cherry shell, and its amount was 1.26%. The presence of sour cherry shell increased the bio‐composite resistance to moisture absorption, but the addition had little effect on the thermal process properties of polyethylene. Vapor permeability in sour cherry shell / polyethylene bio‐composites, which was a significant difference between samples with sour cherry shells (2.5%–7.5%) and samples without sour cherry shells. Results indicated that polyethylene/sour cherry shell composites could be used to replace polyethylene in application such as stretch film, shrink film, and bags of fruit.

## Graphical abstract

Polyethylene/sour cherry shell bio‐composites could be used to replace polyethylene in application such as stretch film, shrink film and bags of fruit. valorization of fruits waste and increasing biodegradability and environmental issues was the objectives of this research.

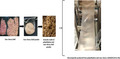

## 1 INTRODUCTION

The use of petroleum‐based synthetic polymers in packaging plays an important role in every aspect of our daily lives. However, the continuous increase in the use of petroleum polymers due to nondegradability causes environmental problems, as well as increasing dependence on oil, increasing the cost of raw materials )Salwa et al.,^2019^). According to the statistics of the Food and Agriculture Organization, about 1.2 million tons of cherries are produced annually in the world, and Iran is the fifth largest producer of cherries in the world with 128,354 tons of this fruit [FAO, 2019]. Sour cherry seeds are used as waste during the processing of this fruit into processed products such as sour cherry jam and sour cherry juice. Industrial processing of cherries produces large amounts of by‐products that are usually destroyed. Kernel products are considered as industrial by‐products after fruit processing as production waste (Yılmaz & Gökmen, 2013). Bio‐composites are compounds that are composed of two or more materials as a matrix and fiber. These materials are stronger on their own when mixed together than separate materials. Fibers obtained from plants can either be from wood or nonwood sources (Mukherjee & Kao, 2011).

Agricultural waste can replace some of the synthetic polymers (Jawaid et al., 2017). Addition three lignocellulosic wastes, that is, almond shell, rice husk and sea grass in poly(3‐hydroxybutyrate), showed that poly(3‐hydroxybutyrate) defects such as brittleness overtime, second crystallization, and physical aging phenomenon as well as production cost were reduced (Sánchez‐Safont et al., 2018). The combination of polypropylene and nutshells of Argan in the production of bio‐composite improved the Young's modulus (Essabir et al., 2013). In the study, two combinations of coconut shell and palm fruit in polyester composites showed better mechanical coconut bio‐composite (Durowaye et al., 2014). The use of *Mimusops elengi* seed shell powder (5%) as bio‐filler in a polypropylene was indicated good dispersion and adhesion which have application in rigid packaging, door panel, etc (Muniyadi et al.,^2018^). Ashori and Nourbakhsh (2010) found that because the bond between the fiber and the matrix, the tensile strength and modulus of the composite improved. Prithivirajan et al., (2015) added rice husk to the epoxy matrix to investigate the effect of this biomaterial on mechanical and thermal properties of bio‐composite. The results showed that the presence of rice husk increases the modulus of elasticity and tensile strength of the epoxy matrix and reduces elongation to failure. Chevalia et al., (2015) added high amounts of hemp to about 10%–30% of the polylactic acid matrix to make its mechanical properties and production economically viable. Qian et al., (2018) prepared bio‐composites from polylactic acid and bamboo cellulose nano‐whiskers and studied their structure and mechanical behavior. The addition of fibers as a biological compound increases the hardness and mechanical properties.

The aim of this research was to evaluate the polyethylene (PE) bio‐composites properties reinforced with different concentration of sour cherry shell powder (SCS). Processability, tensile properties, water absorption, water vapor transmission rate, thermal and morphological properties of polyethylene, and sour cherry shell powder bio‐composites were investigated.

## 2 MATERIALS AND METHODS

### 2.1 Materials

Low‐density polyethylene (with melt index characteristics of 2 g/10 min and density of 0.92 g / cm^3^), linear low‐density polyethylene (with melt index characteristics of 0.9 g / 10 min and density of 0.923 g / cm^3^), and maleic anhydride polyethylene (with melt index characteristics of 2 g /10 min and density of 0.91 g / cm3) were purchased from Krangin company (Iran). Sour cherry seed was supplied from Shana Food Co. (Karaj, Iran).

### 2.2 Bio‐composite preparation

Sour cherry shells were dried in the shade at ambient temperature. After breaking the seed, the separated shells were crushed and powdered with a blender (Moulinex, Spain) and sifted with a 325 U.S. MESH sieve. According to the (Table 1), PE mix (as a matrix contains 83% of low‐density polyethylene, 13% of linear low‐density polyethylene, and 4% of maleic anhydride polyethylene ) and SCS (0%–7.5%) were extruded ( at 165°C, pressure of 85 bar and screw speed of 100 rpm into a double‐helical extrusion machine (ZSK25, Germany). Then, the bio‐composite was prepared using a single‐screw extruder with blowing die film (HAK, Germany) at a temperature of 140 to 170°C with a screw speed of 37 rpm and tensile speed of 100 m / min. The calculated inflation rate was 500%.

**TABLE 1:** Formulation of polyethylene /sour cherry shell (SCS) bio‐composite

Sample code	Composition (%)	
	PE	SCS
PE/SCS—0%	100	0
PE/SCS—2.5%	97.5	2.5
PE/SCS—5%	95.0	5.0
PE/SCS—7.5%	92.5	7.5

### 2.3 Mechanical testing

Tensile test was performed using Universal Testing Machine (model 1,432, China) at a speed of 5 mm/min according to ASTM D638. The composite sheet was cut into dumbbell‐shaped specimens using a dumbbell cutter (Leader Technology Scientific (M) Sdn. Bhd., Balakong, Malaysia). For each composition, 3 dumbbell‐shaped samples (were prepared 20*20 cm^2^) were cut and labeled. The thickness of each specimen had an average thickness of 1.00 mm and was measured using digital thickness gages (JD200, Checkline). The film was subjected to a 500 N load cell, 1,000 mm of extension range, with 26 mm of gauge length, and a crosshead speed of 5 mm/min until the specimen fractured. All tests were conducted at room temperature, and the average values for the tensile strength and elongation at the break of the 3 repeated specimens were summarized for each composition from the stress–strain curve.

### 2.4 Moisture absorption

Moisture absorption of bio‐composite was analyzed based on Angles and Dufresne (2000) method. Samples of bio‐composite with dimensions of (20 × 20) mm^2^ were prepared and placed in a desiccator containing calcium sulfate with relative humidity (RH) 0% for 24 hr. After initial weighing, the samples were transferred to a desiccator containing a saturated solution of calcium nitrite at RH = 55% and placed at a temperature of 20–25°C. Then, the weight of the samples was measured at different times until reaching a constant weight and the amount of moisture absorption was calculated from the following Eq. 1. (This test was repeated three times for each sample).

Moisture absorption (%) _=_ $\frac{\mathrm{Wt}‐\mathrm{Wo}}{\mathrm{Wo}}$ ×100% (1).

W_t_ : Sample weight after time t in RH=% 55.

W_o_: The initial weight of the sample in RH = 0%

### 2.5 Water Vapor Transmission Rate

Water vapor transmission rate (WVTR) was measured by Javanmard (2008) method. In summary, the bio‐composite was tested on a cup containing 12 ml of distilled water. Within 2 hr, steady‐state conditions were assumed to have occurred. The cells were stored in a temperature and humidity controlled room (a/c unit supplied by Denco Ltd., Herts.UK; conditions: 50 ± 5 percentage relative humidity, 23 ± 2°C), and a fan set at an air velocity of 154 mm/min was placed over the cells to ensure uniform movement of air. Eight weight measurements were then recorded over a 24 hr period with intervals of greater than 1.5 hr between readings. At least three replicates of the film samples were tested.

The WVTR was calculated according to Eq. (2).

WVTR = W×X/A (2).

where WVTR is water vapor transmission rate (g H2O mm cm ^−2^), x is the average thickness of the film (mm), and A is the permeation area (cm^2^).

### 2.6 Thermal Properties

The samples were subjected to thermal analysis (differential scanning calorimetry) by a Toledo machine (SDTA 851, Switzerland). The rate of temperature increase was selected linearly at 20°C /min. The atmosphere used was nitrogen gas, and the applied temperature was from ambient temperature up to 500°C. The results of thermal analysis were expressed in a table (4).

### 2.7 Morphological analysis

The tensile fracture surfaces of PE/SCS bio‐composites with filler contents of 0, 2.5, 5, and 7.5% were examined with a scanning electron microscope (*SEM*) (model WEGA‐II TESCAN, Czech Republic). Samples surface to avoid electric charge during experiments were coated with gold for 10 nm and analyzed at 15 keV.

### 2.8 Statistical analysis

Results were expressed as the mean and standard deviation of three independent replicates. All the data were statistically analyzed using one‐way analysis of variance (ANOVA) through Duncan post hoc at *p* <.05. All of the statistical analyses were performed using Minitab software version 16.

## 3 RESULTS AND DISCUSSION

### 3.1 Mechanical testing

**FIGURE 1:**
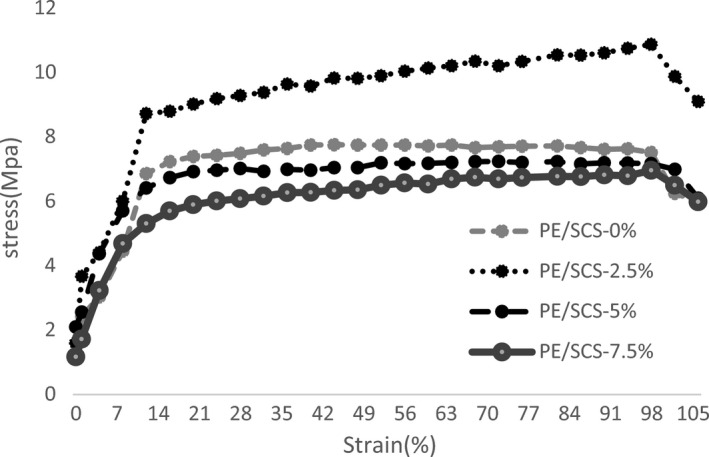
Tensile strength of (polyethylene/sour cherry shell) PE/SCS bio‐composites

Figure 1 shows the stress‐to‐strain diagram from the tensile test for four samples, in which the vertical axis represents the stress and the horizontal axis shows the strain. Tensile strength in the reinforced polymer containing 2.5% of sour cherry shell powder was the highest value. This resistance decreased with increasing amount of sour cherry shell powder, and the lowest tensile strength was seen in the composite containing 7.5% of filler. Due to the curved slope the stress–strain curve, the reinforced polymer containing 2.5% of sour cherry shell had the highest elastic modulus (Table. 2). The effect of increasing sour cherry shell powder was loading on the tensile strength of PE/SCS composites. Tensile strength of PE/SCS composites increased gradually with the increasing of the SCS powder loading to 2.5 wt.% and reduced slightly at a high SCS loading of 5 wt.%. Similar results have been reported for the effect of filler loading in bio‐composites mechanical properties, indicating an increase in composite stiffness and fracture toughness (Fu et al., 2008). Tensile strength for polypropylene composite reinforced with Argan nuts shell was improved with increasing particle loading compared to neat polypropylene (Essabir et al., 2013). Muniyadiet al. (2018) investigated mechanical properties of polypropylenep/*Mimusops elengi* seed shell bio‐composites. Their reports indicated that the presence of up to 5% by weight of *Mimusops elengi* seed shell increased elastic modulus and tensile strength.

**TABLE 2:** Mechanical properties of polyethylene / sour cherry shell bio‐composite (PE/SCS)

	PE/SCS‐0%	PE/SCS‐2.5%	PE/SCS‐5%	PE/SCS‐7.5%
Stress at yield (MPa)	7.7 ±0.02^a^	9.2 ±0.09^b^	7.1 ±0.09^c^	6.1 ±0.02^d^
stress at breaking point (MPa)	9.4 ±0.04^a^	13.2 ±0.05^b^	8.2 ±0.08^c^	7.1 ±0.07^d^
Elongation at break point (%)	317 ±0.05^a^	494 ±0.07^b^	339 ±0.04^c^	189 ±0.3^d^
Elastic modulus (MPa)	6.86 ±0.07^a^	8.73 ±0.02^b^	6.74 ±0.04^c^	5.31 ±0.06^d^

Note:

The data mean 3 replicates (standard deviation ± mean), and different letters in each row indicate the significant effect of adding sour cherry shell powder (0,2.5,5,7.5%).(*p* <.05)

**FIGURE 2:**
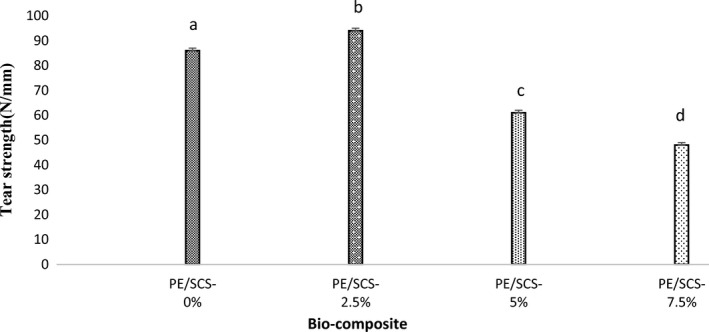
The effect of polyethylene / sour cherry shell bio‐composite (PE/SCS) sheets on tear strength (results are reported as mean ± standard deviation, and the averages shown in each column with different letters are significantly different)

Figure 2 shows the evolution of tear strength of bio‐composites with different contents of sour cherry shell powder loading. Increasing of sour cherry shell powder to the composition increased tear strength and made the composite more durable. The 2.5% loading of sour cherry shell powder increased the tear strength, but in the samples with 5 and 7.5%, tear strength has decreased. In fact, in samples with high loading of sour cherry shell powder particles, we see the formation of areas that finally, it leads to a decrease in the tear strength of the material. The highest tear strength was in bio‐composite containing 2.5% SCS powder loading. This is due to the connection between the polyethylene chain and the sour cherry shell powder. At higher percentages, reinforced polymers tear strength also decreases due to reduced integrity and poor connections (Karger‐Kocsis, 1995). Dos Santos et. al. (2018) observed that the addition of up to 13 wt% of mango's seed shell filler to HDPE, the yield and ultimate stress values were close to those of the neat polymer. At higher fiber content (20 wt %), these properties tend to decrease.

Table 2 shows the mechanical properties of bio‐composites with different contents of sour cherry shell powder loading. The yield point actually indicates the end of the elastic region of the material. In the designs, the yield point is important because the strength of the part in plastic deformation will be significantly damaged. Addition 2.5 wt % of sour cherry shell powder particles increases the breaking point and elongation at the breaking point, which is due to the improved adhesion of sour cherry shell particles to the background (Table 2). In a higher percentage of sour cherry shells powder content in composites, a decrease in the breaking point and elongation at the breaking point were observed, which can be due to the agglomeration of sour cherry shell particles in the matrix. The movement of polypropylene chains as the matrix background increases the elongation at the breaking point. As the sour cherry shell particles increase, agglomeration and voids are created, which weakens the polyethylene chains and reduces the percentage of elongation at the breaking point. Table 2 illustrates the elongation modulus of PE/SCS composites at different SCS powder loading. E‐modulus or tensile modulus refers to the stiffness of composites and its resistance to fracture when stress is applied. The enhancement of E‐modulus can be related to the reduction in segmental mobility of polyethylene chains due to restriction of the SCS particles. Furthermore, good interfacial adhesion between SCS and PE further reduced the mobility of PE chains. A similar observation was reported by Alias et al., (2017). The result showed that enhancement of E‐modulus of polyvinyl alcohol/palm kernel shell powder blends was due to the reduction in segmental mobility of polyvinyl alcohol network in the presence of palm kernel shell powder. Hussein et al., (2011) was found that the addition of egg shell powder to the HDPE led to a decrease in tensile strength, modulus of elasticity, and shore‐D hardness.

SCS powder showed great potential to be used as a new bio‐filler in the PE matrix as depicted from the enhanced tensile strength, tear strength, elongation at break point, stress at breaking point, and stress at yield of PE/SCS composites as compare to neat polymer (PE/SCS—0%).

### 3.2 Moisture absorption

**FIGURE 3:**
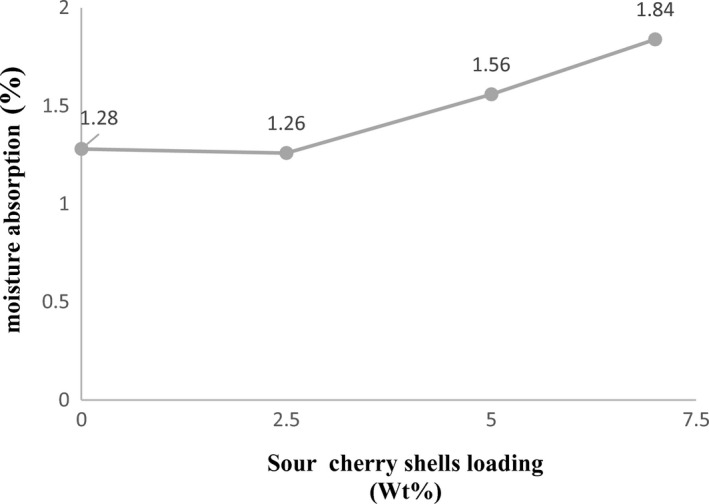
Moisture absorption of polyethylene / sour cherry shell powder bio‐composite (PE/SCS)

Figure 3. shows the effect of loading percentage of sour cherry shell powder on moisture absorption of reinforced PE composites. The moisture absorption rate in the bio‐composite sample containing 2.5% of sour cherry shell is less than the control sample. The moisture absorption rate of the control sample was 1.28%, while this rate was calculated to be 1.26% for bio‐composites containing 2.5% of sour cherry shell powder. Composite contain 2.5% sour cherry shell powder may be led to a decrease in the porosity level and, hence, the low water absorption level of the composites. The presence of hydroxyl groups may lead to moisture absorption by the PE/SCS composites. Thus, water absorption measurement was carried out to determine the water absorption capacity of PE/SCS composites as compared to control sample. This indicates that the presence of sour cherry shell increases the bio‐composite resistance to moisture. As the percentage of sour cherry shell increases to 7.5%, moisture absorption increases. This result shows that adding sour cherry shell to a certain extent can improve the moisture barrier property and will have the opposite effect in higher percentages. Vilaseca et al., (2007) reported similar results of the effect of hemp fibers on reducing the moisture absorption of starch film. The properties of luffa fiber‐treated composites showed that the reduction in water absorption was due to better adhesion between the fiber and the matrix (Demir et al., 2006). Hussein et al., (2011) concluded that composites (high‐density polyethylene/egg shell powder) with higher filler content show more water absorption.

### 3.3 Water Vapor Transmission Rate

Table 3 shows the mean of water vapor transmission rate in sour cherry shell / polyethylene bio‐composites. There was a significant difference between bio‐composites loaded with sour cherry shells powder (2.5%–7.5%) and samples without sour cherry shells. The results showed that the addition of sour cherry shells to PE matrix reduced water vapor transmission rate. Alias et al., (2017) showed that by adding palm kernel shell powder, water vapor permeability in polyvinyl alcohol composites was reduced. This is due to the structure and size of the palm kernel shell powder and the smaller distance between the chains.

**TABLE 3:** Water vapor transmission of polyethylene (PE) /sour cherry shell (SCS) Bio‐composite

Sample code	WVTR (g H2O mm cm ^‐2^)
PE/SCS—0%	2.10 ±0.02^a^
PE/SCS—2.5%	1.50 ±0.07^b^
PE/SCS—5%	1.61 ±0.1^b^
PE/SCS—7.5%	1.69 ±0.08^b^

Note:

Values quoted are mean values ± standard deviations of results for three experiments. Means with the same name are not significantly different. (*p* <.05)

### 3.4 Thermal Properties

Table 4 illustrates the DSC results for the bio‐composite containing 0 to 7.5% SCS powder. As the percentage of sour cherry shells in the composite increases, the melting temperature and crystallization temperature increase. Enthalpy of melting and degree of crystallinity also showed a decreasing but slow trend with increasing containing of sour cherry shell powder. As the SCS powder increased above 2.5 wt.%, the segmental mobility of the PE chains reduced due to stiffening in the PE network, which gradually hinders the rate of crystallization; therefore, the T_c_ and T_m_ decreased slightly and resulted in the enthalpy of melting and degree of crystallinity gradually reduced. The *SEM* morphology also confirmed the presence of SCS powder agglomerates at PE/SCS—7.5%, which could inhibit the crystallization rate and result in a reduction in the degree of crystallinity. Muniyadi et al., (2018) showed that the addition of *Mimusops elengi* seed powder to polypropylene has little effect on thermal properties.

**TABLE 4:** Thermal characteristic of polyethylene / sour cherry shell bio‐composite (PE/SCS)

parameter	SCS loading			
	PE/SCS‐0%	PE/SCS‐2.5%	PE/SCS‐5%	PE/SCS‐7.5%
Melting temperature, T_m_ (°C)	154.34±0.04	147.66±0.05	157.44±0.07	170.44±0.03
Crystallization temperature, T_c_(°C)	92.25±0.06	78.70±0.04	81.38±0.05	98.33±0.09
Enthalpy of melting, ΔH_m_ (J/g)	76.70±0.07	75.80±0.09	74.6±0.2	73.86±0.05
Degree of crystallinity, X^m^_c_(%)	45.89±0.02	44.96±0.06	43.46±0.03	42.97±0.04

### 3.5 Morphological analysis

**FIGURE 4:**
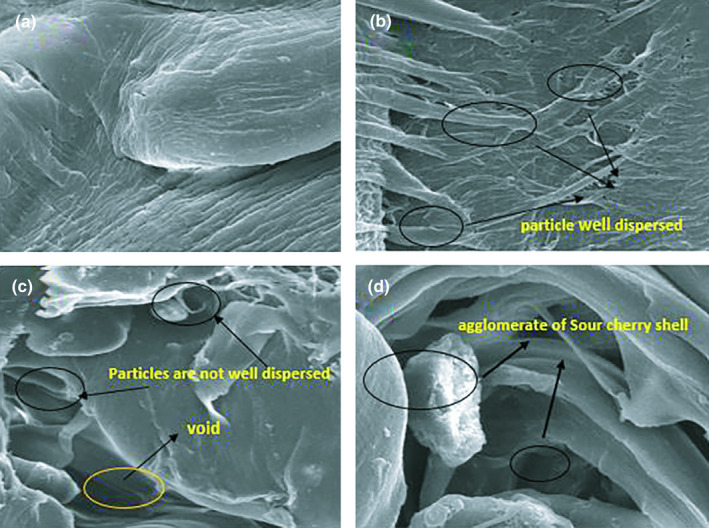
*SEM* micrograph of polyethylene / sour cherry shell bio‐composite (PE/SCS ) at 500x magnification (a) PE/SCS—0%, (b) PE/SCS—2.5%, (c) PE/SCS—5%, and (d) PE/SCS—7.5%

*SEM* micrographs of the polyethylene/sour cherry shell powder composites surface are shown in Figure 4. In Figure 4a, the film sheet containing no sour cherry shell powder shows a uniform surface. Figure 4b shows composite film which containing 2.5% sour cherry shell powder and the sour cherry shell powder attached to the polypropylene matrix. The presence of 2.5% by weight of sour cherry shell powder in the bio‐composite composition has improved the background due to the filling of the holes in the polymer matrix. The effect of bio‐composites as fillers in bio‐composites containing other agricultural wastes has also been reported (Qian et al., 2018). Also in this figure, a relatively good distribution of sour cherry shell powder particles in the polymer sheet was observed. Figure 4c shows a composite film containing 5% by weight of sour cherry shells powder. The particles are not well dispersed, and empty cavities are seen in the polymer matrix. Figure 4d shows composite containing 7.5% by weight of sour cherry shells powder. Polymer reinforced with high concentration of sour cherry shells powder (7.5%), causing agglomeration of particles in the composite film. This agglomeration reduces tension and elongation at the breaking point. According to Muniyadi *et al*.(2018), the aggregated particles of *Mimusops elengi* shell powder in the polypropylene matrix act as stress‐sensitive points and reduce the tensile strength and mechanical properties of polymer composites. In addition, the results of the present study are agreement with the results of Ismail and Mathialagan (2011) that mention agglomeration of bentonite particles in bentonite‐filled ethylene–propylene–diene monomer (EPDM) composites restricts the movement of rubber chain to transfer the applied load and resulting in more rigid composites which consequently leads to reduced elongation at break.

## 4 CONCLUSION

In this work, polyethylene‐based (low‐density polyethylene 83%, linear low‐density polyethylene 13%, and maleic anhydride polyethylene 4%) bio‐composites reinforced with sour cherry shell powder at 0, 2.5, 5, and 7.5 wt % contents were prepared by melt blending in extruder. Addition 2.5% of sour cherry shell powder to polymer matrix improved mechanical and moisture absorption properties of the Bio‐composite film compared to film without sour cherry shell powder. The presence of sour cherry shell improved the tensile properties of the composite by 26%, too. Sour cherry shell powder loading reduced the water absorption in the composites at 2.5% wt % and increased at 5 and 7.5 wt % contents. Morphological evaluation of the bio‐composite showed that the sour cherry shell powder was well bonded to the matrix field.

The results of the present study showed that the developed bio‐composites reinforced with sour cherry shell waste could be a new approach to use the value‐added agricultural waste and reduce environmental pollution and less use of oil resources polymers. Based on the results, it is suggested that these composites can be used in the food industry such as stretch film, shrink film, and bags of fruit.

## CONFLICT OF INTEREST

The authors declare that they do not have any conflict of interest.

## ETHICAL STATEMENT

Ethics approval was not required for this research.

## Data Availability

Research data are not shared.
